# Comparative Study of Dezocine and Ketorolac Tromethamine in Patient-Controlled Intravenous Analgesia of Laparoscopic Cholecystectomy

**DOI:** 10.3389/fsurg.2022.881006

**Published:** 2022-04-25

**Authors:** Yidan Ying, Shuke Fei, Zhiying Zeng, Xiaoyong Qu, Zemin Cao

**Affiliations:** ^1^Department of Pharmacy, Hengyang Medical School, The Second Affiliated Hospital of South China, University of South China, Hengyang, China; ^2^Department of Hepatobiliary and Pancreatic Surgery, Hengyang Medical School, The Second Affiliated Hospital of South China, University of South China, Hengyang, China; ^3^Department of Anesthesiology, Hengyang Medical School, The Second Affiliated Hospital of South China, University of South China, Hengyang, China

**Keywords:** laparoscopic cholecystectomy, dezocine, ketorolac tromethamine, patient-controlled intravenous analgesia, sedation

## Abstract

**Purpose:**

This study aimed to observe the application value of dezocine and ketorolac tromethamine in patient-controlled intravenous analgesia (PCIA) of patients undergoing laparoscopic cholecystectomy (LC).

**Methods:**

A total of 154 patients who underwent LC surgery in our hospital and received PCIA after surgery from September 2020 to September 2021 were selected, they were divided into group A (*n* = 77) and group B (*n* = 77). Group A was given dezocine and group B was given ketorolac tromethamine. The analgesia, sedation, comfort, and adverse reactions of the two groups were closely observed at 4, 8, 12, and 24 h after surgery.

**Results:**

At 4, 8, 12, and 24 h after surgery, the visual analog scale scores in group B were lower than those in group A (*P* < 0.05). At 4, 8, 12, and 24 h after surgery, the Ramsay scores in group B were higher than those in group A (*P* < 0.05). At 4, 8, 12, and 24 h after surgery, there was no significant difference in Bruggrmann comfort scale scores between the two groups (*P* > 0.05). There was no significant difference in the incidence of adverse reactions between the two groups (*P* > 0.05).

**Conclusion:**

Both dezocine and ketorolac tromethamine have high clinical application value in patients who underwent LC surgery and received PCIA, with higher patient comfort and fewer adverse reactions. But compared with dezocine, ketorolac tromethamine can achieve better sedative and analgesic effects, which is worthy of clinical promotion.

## Introduction

Laparoscopic cholecystectomy (LC) has the advantages of short surgery time, good surgical vision, less trauma, less blood loss, easy recovery, and low infection rate, among others. It has been favored and accepted by clinicians and patients and has become the routine surgery of cholecystectomy (1). However, after LC, surgical trauma and pneumoperitoneum will lead to different degrees of physiological pain and discomfort in patients after surgery. This will cause a series of strong stress reactions in the body and may lead to fear, anxiety, irritability, and other adverse emotions, as well as respiratory, circulatory, endocrine, and other dysfunction of the body. These circumstances thus increase the disease burden of patients, slow down the recovery speed after surgery, and lower the quality of life (2, 3). According to the research report, the incidence of postoperative incision pain in patients who underwent LC is as high as 79.2%, and it mainly occurs within 24 h after surgery, which is not conducive to the physical and mental health of patients (4). Therefore, the analgesia and sedation for patients after LC has become an important link for patients to get through the postoperative pain period smoothly.

Relieving postoperative pain of patients undergoing LC is one of the important tasks for clinicians. At present, patient-controlled intravenous analgesia (PCIA) after LC has been widely used in clinics. PCIA refers to when patients feel pain, they can adjust the timing and dosage of intravenous injection of painkillers according to their needs, so as to meet the patient's analgesic needs (5). This method is easy to operate, has an obvious analgesic effect, and embodies the consciousness of patients' active participation in analgesia. Moreover, the injection dose of painkillers is within the range set by doctors, and it is safe (6). PCIA can relieve patients' pain, promote early recovery of patients, and reduce economic burden and medical disputes (7).

In recent years, a variety of analgesic drugs have been continuously and widely developed in the PCIA field. Dezocine and tromethamine ketorolac are both favored for PCIA in clinics. Both of them have certain analgesic effects, but there is limited clinical research on the difference of analgesic effects between these two analgesic methods. In this study, 154 patients who underwent LC surgery and were given PCIA after surgery were selected as the research object. We investigated the analgesic and sedative effects, comfort, and safety of dezocine and tromethamine ketorolac for PCIA.

## Materials and Methods

### Research Object

In this study, 154 patients who underwent LC surgery in our hospital and received PCIA after surgery from September 2020 to September 2021 were selected, they were divided into group A (*n* = 77) and group B (*n* = 77). (1) Inclusion criteria were: compliance with surgical indications; normal mental and cognitive function; and complete case data. (2) Exclusion criteria were: already received second surgery; used drugs within 24 h of surgery that could affect the efficacy of the study drug; important organ dysfunction; drug dependent and/or drug addict; with severe immune system diseases; with severe hypertension and diabetes; and allergic to research drugs.

### Research Methods

Patients in both groups were not given analgesia treatment before surgery. Patients were treated with LC, and the venous channel was established. Induction of anesthesia in the patient was carried out using midazolam 1.5 mg/kg, sufentanil 0.3 μg/kg, and rocuronium 0.6 mg/kg. Then, tracheal intubation anesthesia was administered using sevoflurane, sufentanil 0.15–0.3 μg/kg, and propofol 4 with a concentration of 1–2% during surgery. In the process of using PCIA, patients and their families should be informed of the correct use of analgesic pumps, including how to make analgesic score, how to give medicine by pressing, common side effects, and so on. After surgery, the two groups were connected with the patient-controlled analgesia pump, and all patients were given sufentanil 2 μg/kg. On this basis, group A was given dezocine 0.3 mg/kg, diluted with 100 ml normal saline, and intravenously pumped. Group B was given ketorolac tromethamine 2 mg/kg, diluted with 100 ml normal saline, and intravenously pumped. The infusion rate of the two groups was 2 ml/h, the single self-controlled pressing dose was 2 ml, and the locking time was 15 min. The duration of analgesia was individualized according to the patient's needs.

### Observation Index

The analgesia, sedation, comfort, and adverse reactions of the two groups were closely observed at 4, 8, 12, and 24 h after surgery, and detailed records were made. (1) Analgesia: the visual analog scale (VAS) score was used. In brief, we took a 10 cm scale ruler and asked the patient to mark a scale that can represent their pain degree on the top using a scale of 10 points, with 0 point being painless and 10 points being severe pain. VAS score was assessed by medical staff who were unaware of the patient's condition. (2) Sedation: the Ramsay score was used with the following indicators: 1 point: Sober but anxious; 2 points: Quiet cooperation; 3 points: Sleepy but awake; 4 points: Shallow sleep could wake up; 5 points: Deep sleep but slow response; and 6 points: Deep sleep, no response to call. (3) Comfort: the Bruggrmann comfort scale (BCS) was used with the following scores: 0 point: Persistent pain; 1 point: Painless when quiet, heavy pain when breathing deeply or coughing; 2 points: Painless when quiet, slightly painful when breathing deeply or coughing; 3 points: Painless breathing; 4 points: Cough or other actions are painless; and 5-6 points: Excessive sedation. (4) Adverse reactions were recorded whether there were any adverse reactions such as nausea, vomiting, dizziness, dry mouth, skin pruritus, and urinary retention within 3 days after surgery in both groups.

### Statistical Methods

For statistical analysis, SPSS 22 (Armonk, NY: IBM Corp.) was used for data processing. The measurement data conforming to the normal distribution were expressed as mean ± *SD*. Paired sample *t*-test was used for intra-group comparison, while an independent-sample *t*-test was used for inter-group comparison. The counting data were expressed by %, and the χ^2^ test was used for comparison. *P* < 0.05, the difference was statistically significant.

## Results

### General Information

There was no significant difference in gender, age, intraoperative blood loss, and surgery time between the two groups (*P* > 0.05; Table 1).

**Table 1:** Comparison of two groups of general information.

**Group**	**Case number**	**Gender**		**Age(years)**	**Intraoperative blood loss (ml)**	**Surgery time (min)**
		**Male**	**Female**			
Group A	77	42 (54.55%)	35 (45.45%)	45.92 ± 9.36	8.73 ± 1.24	90.35 ± 6.91
Group B	77	39 (50.65%)	38 (49.35%)	46.27 ± 9.50	8.56 ± 1.19	92.11 ± 7.05
*χ^2^/t value*		0.234		0.230	0.867	1.564
*P value*		0.628		0.818	0.386	0.119

### Analgesic Situation

**Figure 1:**
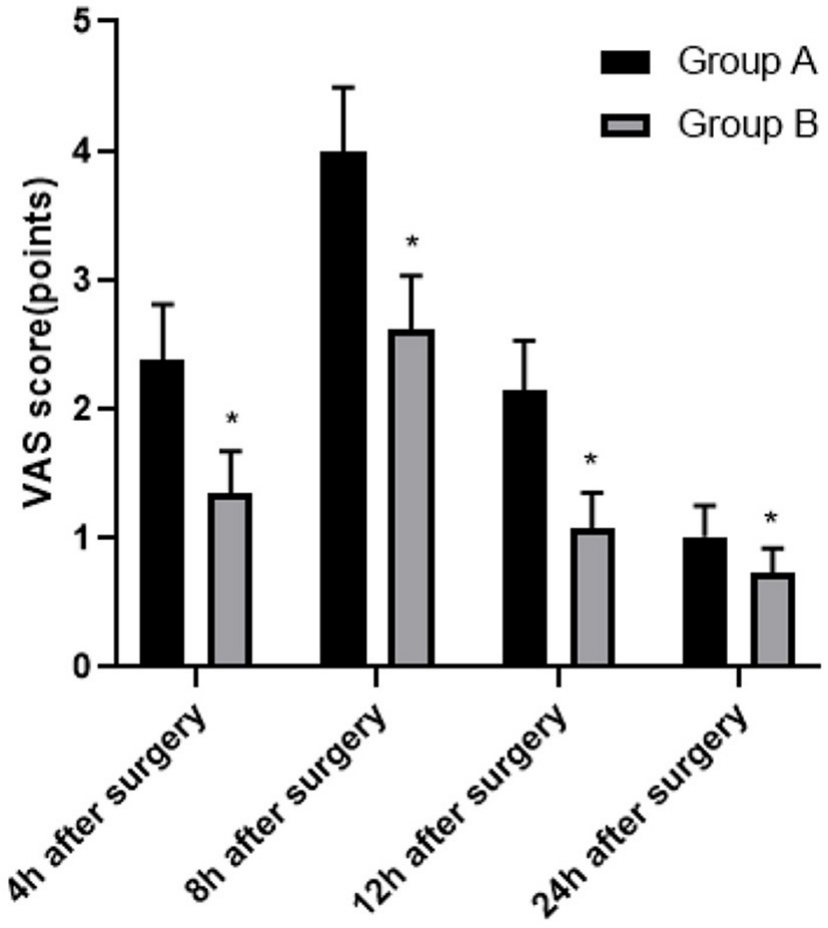
Comparison of the analgesic situation between two groups. Compared with group A, *P < 0.05.

At 4, 8, 12, and 24 h after surgery, the VAS scores in group B were lower than those in group A (*P* < 0.05; Figure 1).

### Sedation Situation

**Figure 2:**
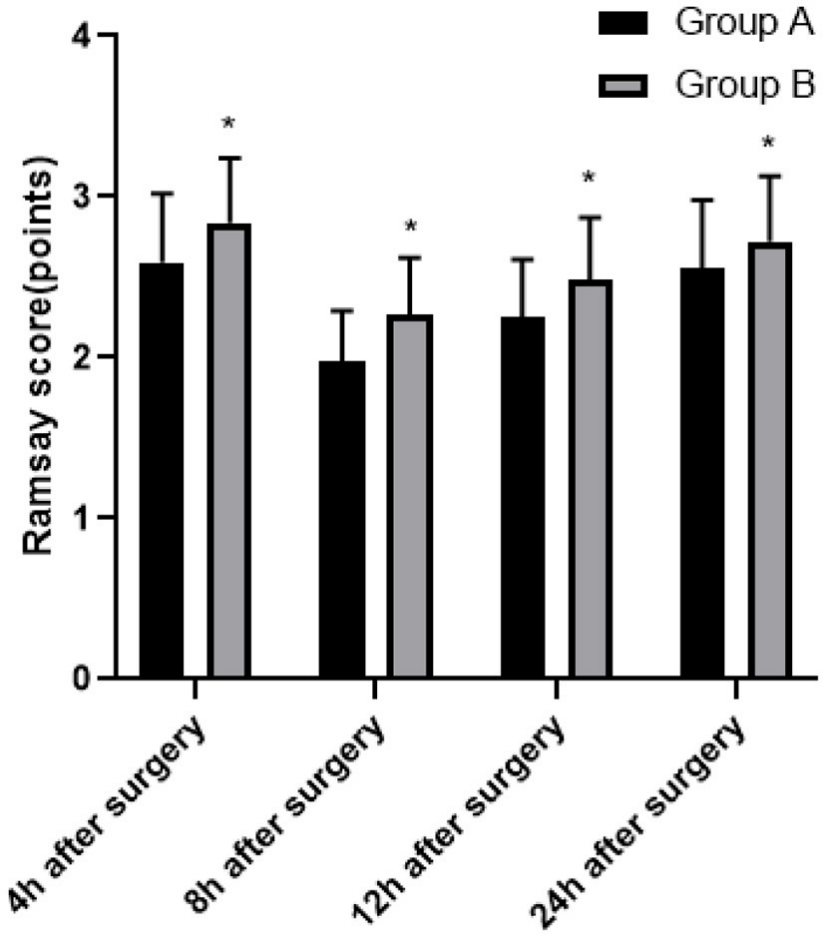
Comparison of sedation situation between two groups. Compared with group A, *P < 0.05.

At 4, 8, 12, and 24 h after surgery, the Ramsay scores in group B were higher than those in group A (*P* < 0.05; Figure 2).

### Comfort Situation

**Figure 3:**
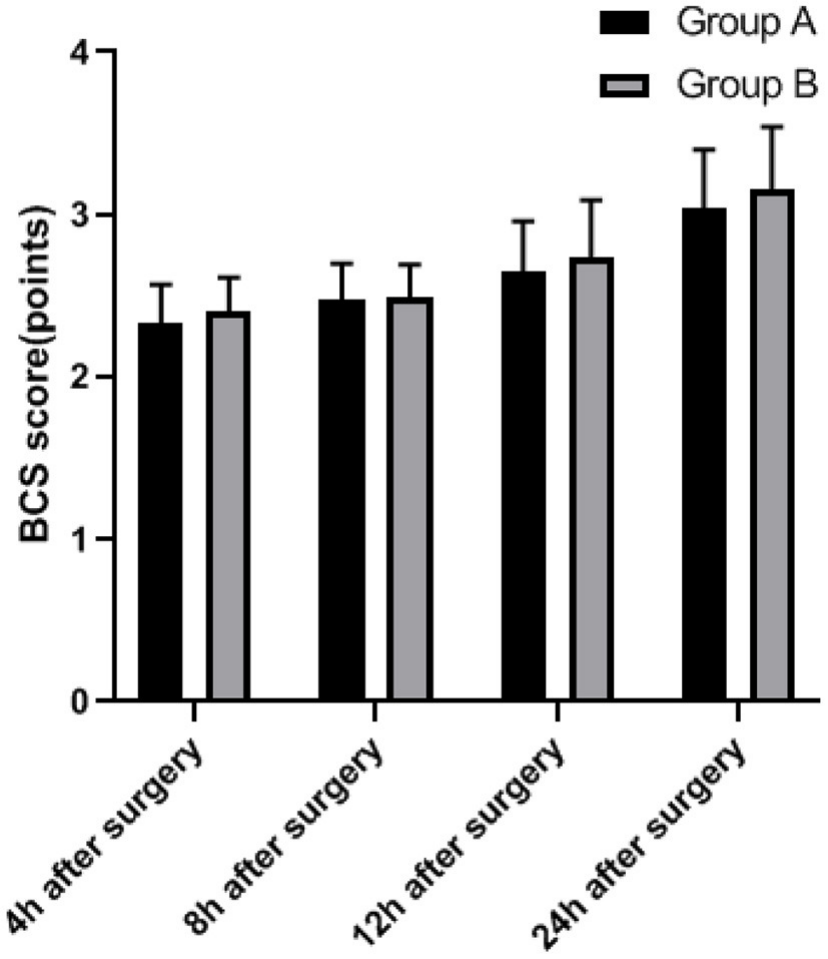
Comparison of comfort between two groups.

At 4, 8, 12, and 24 h after surgery, there was no significant difference in BCS scores between the two groups (*P* > 0.05; Figure 3).

### Adverse Reactions

There was no significant difference in the incidence of adverse reactions between the two groups (*P* > 0.05; Table 2).

**Table 2:** Comparison of adverse reactions between two groups.

**Group**	**Case number**	**Nausea**	**Vomiting**	**Dizziness**	**Dry mouth**	**Skin pruritus**	**Urinary retention**	**Total**
Group A	77	4 (5.19%)	2 (2.59%)	1 (1.30%)	1 (1.30%)	1 (1.30%)	0 (0.00%)	9 (11.69%)
Group B	77	2 (2.59%)	2 (2.59%)	1 (1.30%)	0 (0.00%)	0 (0.00%)	0 (0.00%)	5 (6.49%)
*χ^2^ value*								1.257
*P value*								0.262

## Discussion

Laparoscopic cholecystectomy surgery has a significant positive effect as an effective method for the clinical treatment of cholecystitis, cholecystolithiasis, and other gallbladder diseases (8). However, due to factors such as diaphragm traction and visceral mucosa hypoxia after CO_2_ pneumoperitoneum, patients after LC are prone to pain symptoms, which affects their physical and mental health and prognosis recovery (9). Postoperative pain can inhibit the patient's breathing activity, increase the patient's blood pressure, increase the oxygen consumption of myocardium, inhibit gastrointestinal peristalsis, reduce the body's resistance, and easily cause some negative emotions, thus affecting the prognosis of the disease (10, 11). Therefore, how to effectively relieve postoperative pain of patients is of great significance to the physical and mental health of patients. At present, dezocine and ketorolac tromethamine are widely used in PCIA.

Dezocine is a potent opioid analgesic with both exciting and antagonistic effects (12). On the one hand, dezocine mainly achieves analgesic and sedative effects by exciting K receptors distributed in the brain, spinal cord, and brain stem (13). On the other hand, dezocine is also a μ-receptor antagonist, which has no typical μ-receptor dependence, so it can relax gastrointestinal smooth muscle with fewer adverse reactions (14). At the same time, dezocine can inhibit the activities of endothelin and renin in the body, reduce the release of angiotensin II and inflammatory factors, thereby reducing the excessive stress reaction of patients after LC (15). Dezocine has similar effects to morphine in onset time, action time, and intensity of action, and it is not easy to produce tolerance and can maintain the relative stability of blood volume and hemodynamics (16).

Ketorolac tromethamine is a non-steroidal anti-inflammatory drug with the best analgesic effect at present, and its analgesic effect is 7.5 times that of morphine, which is suitable for short-term treatment of acute moderate, and severe pain (17). It has strong analgesic and sedative effects in many aspects, and the specific mechanisms are as follows: ① Prostaglandin is an important mediator that causes inflammation such as redness, swelling, heat, and pain in the body. Ketorolac tromethamine can reduce the synthesis of prostaglandins by inhibiting the activity of peripheral and central cyclooxygenase, thereby reducing the degree of inflammatory reaction in tissues and relieving the stimulation of inflammatory medium to nerve endings, so as to achieve the analgesic effect (18). ② The medicine can reduce the response of pain nerves to endogenous inflammatory factors, and passivate peripheral nerves in advance to prevent peripheral nerve sensitization, thereby reducing the body's response to stressors, inhibiting sympathetic nerve excitation and thus reducing the level of stress factors, reducing the pain of patients after LC (19). ③ This medicine is not only beneficial to alleviate the wound inflammatory reaction and tissue edema caused by LC, but also affects the synthesis and activity of other nerve active substances and prevents the nerve active substances of nociceptive stimulation from being conducted to the spinal nerve (20). ④ Ketorolac tromethamine can selectively aggregate the pain site, thus increasing the drug concentration, which makes the analgesic effect stronger, the action time longer, and the sedative effect stronger (21). ⑤ Some scholars have found that ketorolac tromethamine may have an effect on the hypothalamic prostate system In addition, its analgesic effect may be related to the synthesis and participation of nitric oxide (22, 23). However, more experiments are needed to confirm this conclusion.

The commonly used multimodal analgesia in clinical practice is sufentanil combined with dezocine. Sufentanil has a longer sedative effect time and less inhibitory effect on respiratory function. Sufentanil has strong lipophilicity and a higher rate of passing through the blood-brain barrier, thereby increasing the binding rate to plasma proteins, subsequently achieving an analgesic effect. The results showed that within 24 h after surgery, the VAS score of group B was lower, the Ramsay score of this group was higher, and there was no significant difference in BCS score and incidence of adverse reactions between group A and group B. We believe that both dezocine and ketorolac tromethamine have high clinical application value in patients who underwent LC surgery and received PCIA, with higher patient comfort and less adverse reactions. But compared with dezocine, ketorolac tromethamine can achieve better sedative and analgesic effects. At present, the common clinical adverse reactions are nausea and vomiting, and serotonin receptor blockers and glucocorticoids are the first-line drugs to deal with nausea and vomiting. If both are ineffective, droperidol can be used. In addition, other possible adverse reactions include dizziness, dry mouth, skin pruritus, and urinary retention, which generally do not require special treatment. It is worth mentioning that there are still some shortcomings in this study, such as the following: the scheme is a single-center study, and hence multi-center research needs to be carried out in the follow-up study. The infusion rates of both groups were 2 ml/h, and only one PCIA infusion rate was used. The PCIA infusion rate should be adjusted individually according to the needs of patients. The doses of dezocine and ketorolac tromethamine have not been classified hierarchically, and we still need to improve the research (24–26).

## Conclusion

Both dezocine and ketorolac tromethamine have high clinical application value in patients who underwent LC surgery and received PCIA, with higher patient comfort and fewer adverse reactions. However, compared with dezocine, ketorolac tromethamine can achieve better sedative and analgesic effects, which is worthy of clinical promotion.

## Data Availability Statement

The original contributions presented in the study are included in the article/supplementary material, further inquiries can be directed to the corresponding author.

## Ethics Statement

The studies involving human participants were reviewed and approved by the Ethics Committee of the Second Affiliated Hospital of South China. The patients/participants provided their written informed consent to participate in this study.

## Author Contributions

YY is the mainly responsible for the writing of the article. SF is mainly responsible for research design. ZZ and XQ are mainly responsible for data analysis. ZC is responsible for the guidance of the entire research. All authors contributed to the article and approved the submitted version.

## Conflict of Interest

The authors declare that the research was conducted in the absence of any commercial or financial relationships that could be construed as a potential conflict of interest.

## Publisher's Note

All claims expressed in this article are solely those of the authors and do not necessarily represent those of their affiliated organizations, or those of the publisher, the editors and the reviewers. Any product that may be evaluated in this article, or claim that may be made by its manufacturer, is not guaranteed or endorsed by the publisher.
